# Subregional Differences in Medium Spiny Neuron Intrinsic Excitability Properties between Nucleus Accumbens Core and Shell in Male Rats

**DOI:** 10.1523/ENEURO.0432-22.2023

**Published:** 2023-05-17

**Authors:** Cristina E. Maria-Rios, Geoffrey G. Murphy, Jonathan D. Morrow

**Affiliations:** 1 Neuroscience Graduate Program, University of Michigan, Ann Arbor, MI 48109; 2 Michigan Neuroscience Institute, University of Michigan, Ann Arbor, MI 48109; 3 Department of Molecular and Integrative Physiology, University of Michigan, Ann Arbor, MI 48109; 4 Department of Psychiatry, University of Michigan, Ann Arbor, MI 48109

**Keywords:** electrophysiology, GABAergic, patch clamp, reward, ventral striatum

## Abstract

The nucleus accumbens (NAc) is known for its central role in reward and motivation (Day and Carelli, 2007; Floresco, 2015; Salgado and Kaplitt, 2015). Decades of research on the cellular arrangement, density, and connectivity of the NAc have identified two main subregions known as the core and shell (Záborszky et al., 1985; Berendse and Groenewegen, 1990; Zahm and Heimer, 1990). Although anatomically and functionally different, both the NAc core and shell are mainly comprised of GABAergic projection neurons known as medium spiny neurons (MSNs) (Matamales et al., 2009). Several studies have identified key morphologic differences between core and shell MSNs (Meredith et al., 1992; Forlano and Woolley, 2010) but few studies have directly addressed how core and shell MSNs differ in their intrinsic excitability (Pennartz et al., 1992; O’Donnell and Grace, 1993). Using whole-cell patch-clamp recordings in slices prepared from naive and rewarded male rats, we found that MSNs in the NAc shell were significantly more excitable than MSNs in the NAc core in both groups. In the shell, MSNs had significantly greater input resistance, lower cell capacitance, and a greater sag. This was accompanied by a lower action potential current threshold, a greater number of action potentials, and faster firing frequency compared with core MSNs. These subregional differences in intrinsic excitability could provide a potential physiological link to the distinct anatomic characteristics of core and shell MSNs and to their distinct functional roles in reward learning (Zahm, 1999; Ito and Hayen, 2011; Saddoris et al., 2015; West and Carelli, 2016).

## Significance Statement

The nucleus accumbens (NAc) is a critical structure in the integration process of reward information necessary to regulate motivated behaviors. It has been divided into two main subregions known as the core and shell. The intrinsic excitability state of medium spiny neurons (MSNs), the main neuronal population of the NAc core and shell, can heavily influence how the NAc encodes and relays reward information. Understanding how the different subregions of the NAc respond to input stimuli by studying their intrinsic excitability is crucial for further dissecting the functional segregation of the NAc subregions in reward learning and ultimately linking it to disorders like addiction and anxiety.

## Introduction

The nucleus accumbens (NAc) is a part of the ventral striatum located within the basal forebrain. Decades of research have identified a fundamental role for the NAc in reward and motivation, making it a crucial structure for understanding numerous neuropsychiatric disorders including addiction, anxiety, depression, and bipolar disorder (Day and Carelli, 2007; Floresco, 2015; Salgado and Kaplitt, 2015). Based on differences in cellular arrangement, density, and connectivity, previous anatomic and histologic studies have divided the NAc into three subregions: core, shell, and rostral pole, where core and shell are indistinguishable (Záborszky et al., 1985; Berendse and Groenewegen, 1990; Zahm and Heimer, 1990). For example, some studies have shown that neurons in the NAc core and shell differ in their morphology with cells in the core having greater total surface area, dendritic branching, and spine density than cells in the shell (Meredith et al., 1992; Forlano and Woolley, 2010). Additionally, core and shell neurons show substantial differences in both afferent and efferent connections with striatal, mesencephalic, hypothalamic, amygdalar, cortical, and hippocampal regions (Záborszky et al., 1985; Berendse and Groenewegen, 1990; Zahm and Heimer, 1990; Heimer et al., 1991; Berendse et al., 1992; Meredith et al., 1992; Britt et al., 2012). Collectively, these subregional differences in NAc anatomy and connectivity, may be responsible for the distinctive functional roles in reward and motivation attributed to the core and shell (Zahm, 1999). These include functional differences in instrumental learning, Pavlovian conditioned approach, reward devaluation, impulsivity, as well as food-seeking and cocaine-seeking behaviors (Day and Carelli, 2007; Floresco, 2015).

Approximately 95% of the neurons in both the core and the shell of the NAc are GABAergic projection neurons known as medium spiny neurons (MSNs; Matamales et al., 2009). Within subregions, many electrophysiological studies have thoroughly characterized the intrinsic excitability properties of MSNs in rodents, including how these vary by sex (Cao et al., 2018; Proaño et al., 2018), estrous cycle (Proaño et al., 2018; Proaño and Meitzen, 2020), and neuronal subtype (Cao et al., 2018; Deroche et al., 2020), how they are modulated by dopamine (O’Donnell and Grace, 1996; Perez et al., 2006; Podda et al., 2010), and substances like cocaine (Kourrich and Thomas, 2009), and how they are altered in models of addiction (Mu et al., 2010; Graves et al., 2015), obesity (Alonso-Caraballo and Ferrario, 2019; Oginsky and Ferrario, 2019), stress, and depression (Francis et al., 2015, 2019). Despite all the anatomic and functional evidence suggesting physiological differences between NAc subregions, very few studies have directly investigated how core and shell MSNs differ with one another in their passive and active intrinsic excitability properties. Previous findings in mice suggest that differences in input resistance between core and shell MSNs may result in greater excitability in shell MSNs (Kourrich and Thomas, 2009). In comparison, studies in rats have suggested very subtle and contrasting subregional differences, leaving uncertainty as to what the physiological differences between core and shell MSNs may be (Pennartz et al., 1992; O’Donnell and Grace, 1993).

For this study, we used whole-cell patch-clamp recordings to conduct a comprehensive electrophysiological analysis of the passive and active membrane properties of MSNs in the NAc core and shell of adult male rats. Since behavioral and environmental enrichment is known to alter intrinsic excitability of certain neuronal subtypes like MSNs in the NAc (Scala et al., 2018) and pyramidal neurons in the hippocampus (Malik and Chattarji, 2012; Valero-Aracama et al., 2015), we not only studied “naive” animals, but also rats that underwent six sessions of unpaired cue/food reward exposures (“rewarded”). The goal of our study is to characterize subregional physiological differences between the core and shell of the NAc in naive rats and explore whether these remain stable in “rewarded” rats that underwent behavioral enrichment.

## Materials and Methods

### Animals

All animal procedures were previously approved by the University Committee on the Use and Care of Animals (University of Michigan, Ann Arbor, MI). Thirty-one adult male Sprague Dawley rats (seven to eight weeks) were purchased from Charles River Laboratories (C72, R04) and housed in pairs. Rats were maintained on a 12/12 h light/dark cycle, and food and water were available *ad libitum* for the entirety of experimentation. Rats were divided into two counterbalanced groups before the study began: Naive (*n* = 13) and Rewarded (*n* = 18). Naive rats remained in their home cages and received no handling before electrophysiological recordings. Rewarded rats were acclimatized to the housing colony for at least 2 d before handling. After behavioral testing the rats remained in their home cages for a baseline period of one to three weeks before electrophysiological recordings.

### Drugs

Isoflurane (Fluriso - VetOne) was administered at 5% via inhalation for inducing anesthesia. Picrotoxin (Tocris Bioscience) was dissolved in artificial CSF (ACSF) to make a 100 μM solution. Kynurenic acid (Sigma-Aldrich) was dissolved in ACSF to make a 5 mM solution.

### Behavioral testing apparatus

Sixteen modular operant conditioning chambers (24.1 cm in width × 20.5 cm in depth × 29.2 cm in height; MED Associates) were used for behavioral enrichment. Each chamber was in a sound-attenuating cubicle equipped with a ventilation fan to provide ambient background noise. Each chamber was equipped with a food magazine, a retractable lever (counterbalanced on the left or right side of the magazine), and a red house light on the wall opposite of the magazine. The magazine contained an infrared sensor to detect magazine entries, and the levers were calibrated to detect lever deflections in response to 10 g of applied weight. Whenever a lever was extended into the chamber, an LED mounted inside the lever mechanism illuminated the slot through which the lever protruded (ABET II Software; Lafayette Instrument).

### Behavioral testing procedure

Rats were not food deprived at any point during experimentation. All rats in the Rewarded group were habituated for 2 d before the start of training. Rats were handled individually and were familiarized with banana-flavored pellets (45 mg; Bio-Serv) in their home cages. On the third day, rats were placed into the test chambers for one pretraining session during which the red house-light remained on, but the lever was retracted. Twenty-five food pellets were delivered on a variable time (VT) 30-s schedule (i.e., one pellet was delivered on average every 30 s, but varied 0–60 s). Next, rats underwent six daily sessions of behavioral training. Each trial during a training session consisted of a presentation of the illuminated lever into the chamber for 10 s and a response-independent delivery of one pellet into the magazine. Both stimuli were on a VT 45-s schedule (i.e., time randomly varied 30–60 s between presentations). The beginning of the next intertrial interval commenced once both the lever and the pellet had been presented. Each test session consisted of 25 trials of random unpaired lever and pellet presentations. All rats consumed all the pellets that were delivered.

### Electrophysiology

#### Slice preparation

Rats were deeply anesthetized with isoflurane (Kent Scientific) and euthanized by decapitation. The brain was rapidly dissected and glued on a platform submerged in an ice-cold oxygenated (95% O_2_/5% CO_2_) cutting solution containing (in mM): 206 sucrose (Sigma-Aldrich), 10 D-glucose (Sigma-Aldrich), 1.25 NaH_2_PO_4_ (Sigma-Aldrich), 26 NaHCO_3_ (Sigma-Aldrich), 2 KCl (Fisher Chemical), 0.4 sodium ascorbic acid (Sigma-Aldrich), 2 MgSO_4_ (Sigma-Aldrich), 1 CaCl_2_ (Sigma-Aldrich), and 1 MgCl_2_ (Sigma-Aldrich). A mid-sagittal cut was made to divide the two hemispheres, and coronal brain slices (300 μm) were cut using a vibrating blade microtome (Leica VT1200). The brain slices were transferred to a holding chamber with oxygenated artificial CSF (ACSF) containing (in mM): 119 NaCl (Sigma-Aldrich), 2.5 KCl (Fisher Chemical), 1 NaH_2_PO_4_ (Sigma-Aldrich), 26.2 NaHCO_3_ (Sigma-Aldrich), 11 D-glucose (Sigma-Aldrich), 1 sodium ascorbic acid (Sigma-Aldrich), 1.3 MgSO_4_ (Sigma-Aldrich), and 2.5 CaCl_2_ (Sigma-Aldrich; ∼295 mOsm, pH 7.2–7.3) at 37°C for 20 min and then room temperature for at least 40 min of rest. The slices were kept submerged in oxygenated ACSF in a holding chamber at room temperature for up to 7–8 h after slicing.

#### Electrophysiological recordings

After at least 1 h of rest, slices were transferred to the recording chamber where they were perfused with oxygenated ACSF (32°C) containing 100 μM of GABA_A_ receptor antagonist, picrotoxin (Tocris Bioscience) and 5 mM of kynurenic acid (Sigma-Aldrich) to block glutamatergic transmission. Recordings from the NAc core and medial shell were done in the same slices which were obtained between +1.00 and +1.70 mm anterior from bregma (Paxinos and Franklin, 2019). Cells were visualized using infrared differential interference contrast (IR-DIC) optics (Microscope: Olympus BX51; Camera: Dage-MIT). Whole-cell current clamp recordings were performed using borosilicate glass pipettes (O.D. 1.5 mm, I.D. 0.86 mm; Sutter Instruments) with a 4–7 MΩ open tip resistance. Pipettes were filled with a potassium gluconate-based internal solution containing (in mM): 122 K-gluconate (Sigma-Aldrich), 20 HEPES (Sigma-Aldrich), 0.4 EGTA (Sigma-Aldrich), 2.8 NaCl (Sigma-Aldrich), and 2 Mg^2+^ATP/0.3 Na_2_GTP (∼280 mOsm, pH adjusted to 7.2 with KOH). Medium spiny neurons were identified based on morphology (medium-sized soma) as well as a hyperpolarized resting potential between −70 and −90 mV and inward rectification. Neurons exhibiting a resting potential out of the desired range, characteristics of fast-spiking interneurons, and irregular firing pattern were excluded. All recordings were obtained using the MultiClamp 700B (Molecular Devices) amplifier and Digidata 1550A (Molecular Devices) digitizer. Data were filtered at 2 kHz, digitized at 10 kHz, and were collected and analyzed using pClamp 10.0 software (Molecular Devices). Recordings were not adjusted for the calculated liquid junction potential of 15.8 mV.

To perform whole-cell recordings, membrane seals with a resistance >1 GΩ were achieved before breaking into the cell. Membrane capacitance (C_m_) and series resistance (R_s_) were compensated under voltage-clamp, and C_m_ was recorded 1 min after breaking in. R_s_ was recorded in voltage-clamp with an average of 31 ± 12 (MΩ) on entry and 29 ± 12 (MΩ; mean ± SD) once the recordings were finished. Firing properties were recorded under current-clamp, and input resistance (R_i_) was monitored online during each sweep with a −100-pA, 25-ms current injection separated by 100 ms from the current injection step protocols. The average R_i_ across all sweeps is reported. Only cells with an R_i_ that remained stable (Δ < 20%) were included in the analysis (Naive: *n* = 51, Rewarded: *n* = 78). All neurons underwent two recording protocols from their RMP to assess firing properties. To study spike number, spike frequency, voltage/current relationships, and sag ratios, neurons were subjected to a step protocol consisting of 500-ms current injections starting from −500 to +500 pA in 25-pA increments. Each sweep was separated by 4 s. Resting membrane potential (RMP) was reported as the average voltage from all sweeps at 5 ms. The number of spikes was determined by counting the number of individual spikes at each current injection. Firing frequency was determined by averaging the frequency (in Hz) between each two spikes for a given current injection. If a neuron reached depolarization block, data for that cell were reported until the current injection before the depolarization block (2.5% of cells in core and 9.8% in shell). The steady-state voltage responses were measured 200 ms from the onset of stimulation for each subthreshold current injection step. Sag ratios were determined by the ratio of the peak voltage at the most hyperpolarized current injection (−500 pA) over the steady-state response. A ratio of 1.00 would represent no sag, and therefore, the greater the ratio, the larger the sag. For voltage/current relationship, the voltage reported is the δ between the steady-state and the baseline voltage 1 ms before onset of stimulation.

To study single action potential (AP) firing properties, neurons received 5-ms current injections in 25-pA increments until a single AP was elicited. Each sweep was separated by 4 s. Current to threshold (pA) was determined as the minimal current injection necessary to induce a single AP. The AP threshold (mV) was defined from 0 mV as the voltage at the AP inflection point. The Δ RMP/AP threshold (mV) was determined by taking the difference between the RMP and the AP threshold determined by the AP inflection point. The AP amplitude (mV) was defined from 0 mV as the voltage at the peak of the AP overshoot. The Δ RMP/AP amplitude (mV) was determined by taking the difference between the RMP and the AP amplitude measured from 0 mV. The Δ AP threshold/AP amplitude (mV) was determined by taking the difference between the AP threshold and the AP amplitude. Finally, AP halfwidth (ms) was defined as the duration of the AP at half the voltage of the peak amplitude.

### Experimental design and statistical analysis

A total of 129 cells from 31 rats were included in the analysis. The naive group had a total of 13 rats, from which 31 cells were recorded from in the core of 13 rats (one to two slices/one to four cells per rat) and 20 cells in the shell of nine of the rats (one slice/one to three cells per rat). The rewarded group had a total of 18 rats, from which 47 cells were recorded from in the core of 18 rats (one to two slices/one to five cells per rat) and 31 cells in the shell of 14 of the rats (one to three slices/one to four cells per rat). A total of 49 cells (Naive: *n* = 25, Rewarded: *n* = 24) were excluded from the analyses of sag ratio and voltage/current relationship curves. For these cells the step protocol ranged from −200 to +500 pA as opposed to −500 to +500 pA. They were excluded to keep the hyperpolarized current injection analysis homogeneous.

All offline analysis of electrophysiological recordings was performed using Clampfit 10.7 (Molecular Devices). Statistical analyses were made using GraphPad Prism 8 (Dotmatics) and SPSS Statistics (IBM) software. RMP, C_m_, R_i_, sag ratio, current to threshold, AP threshold, ΔRMP/AP threshold, AP amplitude, ΔRMP/AP amplitude, ΔAP threshold/AP amplitude, and AP halfwidth were analyzed using two-way ANOVA with subregion (core vs shell) and group (naive vs rewarded) as independent variables. Sidak’s *post hoc* test was used for multiple comparisons. Data are presented as mean ± SEM with each data point representing an individual cell, and significance level was set at *p* < 0.05. Number of spikes, spike firing frequency, and voltage/current relationship curves (−500 to 0 pA, 0 to +100 pA) were analyzed using linear mixed-effects model via restricted maximum likelihood (REML). Fixed effects were set for subregion (core, shell), group (naive, rewarded), current injection (V/I: −500 to 0 pA, 0 to +100 pA; AP: +50 to +400 pA), subregion × group, subregion × current injection, group × current injection, and subregion × group × current injection. Multiple comparisons were made using Sidak’s *post hoc* test. Based on the group × subregion statistical report, planned comparisons using mixed-effects model via REML were done to obtain individual subregion statistics for naive (core vs shell) and rewarded (core vs shell) rats and group statistics for core (naive vs rewarded) and shell (naive vs rewarded). Fixed effects to obtain subregion statistics within groups were set for subregion (core, shell), current injection (V/I: −500 to 0 pA, 0 to +100 pA; AP: +50 to +400 pA), and subregion × current injection. Fixed effects to obtain group statistics within subregion were set for group (naive, rewarded), current injection (V/I: −500 to 0 pA, 0 to +100 pA; AP: +50 to +400 pA), and group × current injection. Data are presented as mean ± SEM with significance level set at *p* < 0.05.

## Results

### NAc MSNs exhibit distinct passive membrane properties in the core versus shell subregions of both naive and rewarded rats

**Figure 1.**
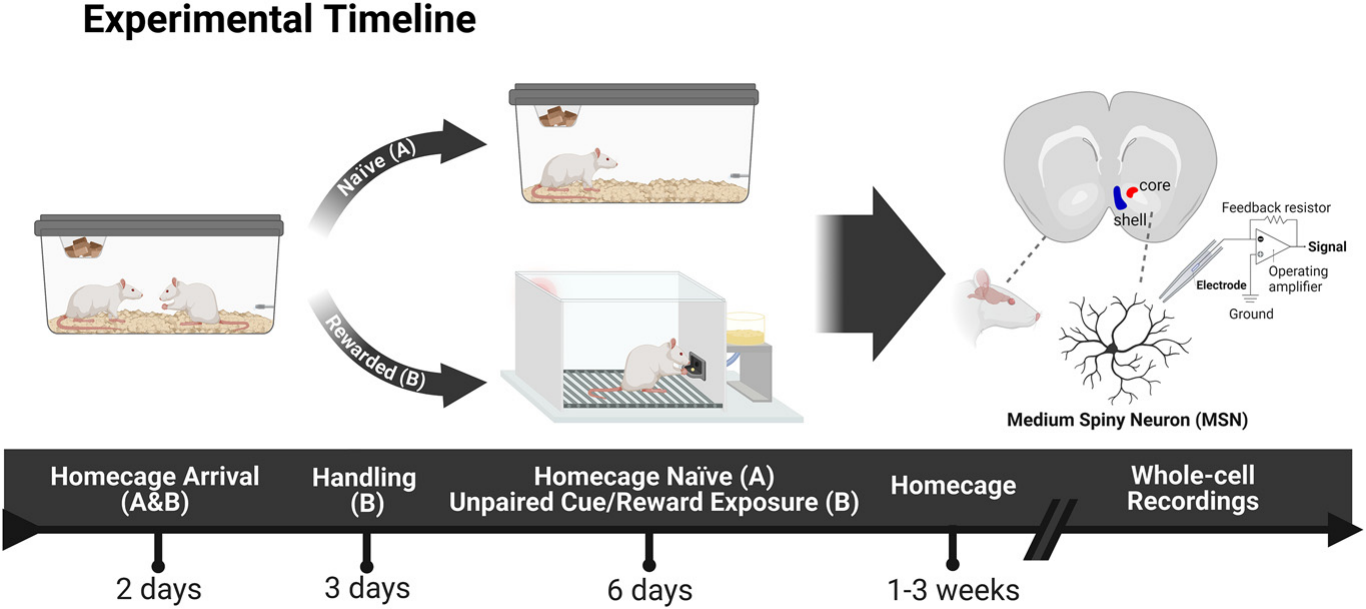
Experimental timeline. All rats were housed in pairs on arrival. The Naive group (***A***) was randomly selected and remained unhandled in their homecages for the entirety of the experiment until electrophysiological recordings were performed. The Rewarded group (***B***) was handled for 2 d after at least 2 d of acclimation to the housing room. They were then exposed to a pretraining session in the behavioral test apparatus where they received 25 pellets into a food-cup over the course of 30 min. For the following 6 d, they underwent a daily behavioral experience in which a neutral lever-cue was presented, and banana food pellet rewards were delivered into a food-cup randomly and independently of one another. Each session consisted of 25 independent trials of lever and reward presentations (ITI: 30–60 s). After the last session of unpaired cue/reward exposures, rats remained in their homecages for a period of one to three weeks. Subsequently, nucleus accumbens slices were prepared for whole-cell recordings of medium spiny neurons in the core and shell subregions. Behavioral responses for Rewarded rats were recorded and can be found in Extended Data Figure 1-1. Created with BioRender.

**Figure 2.**
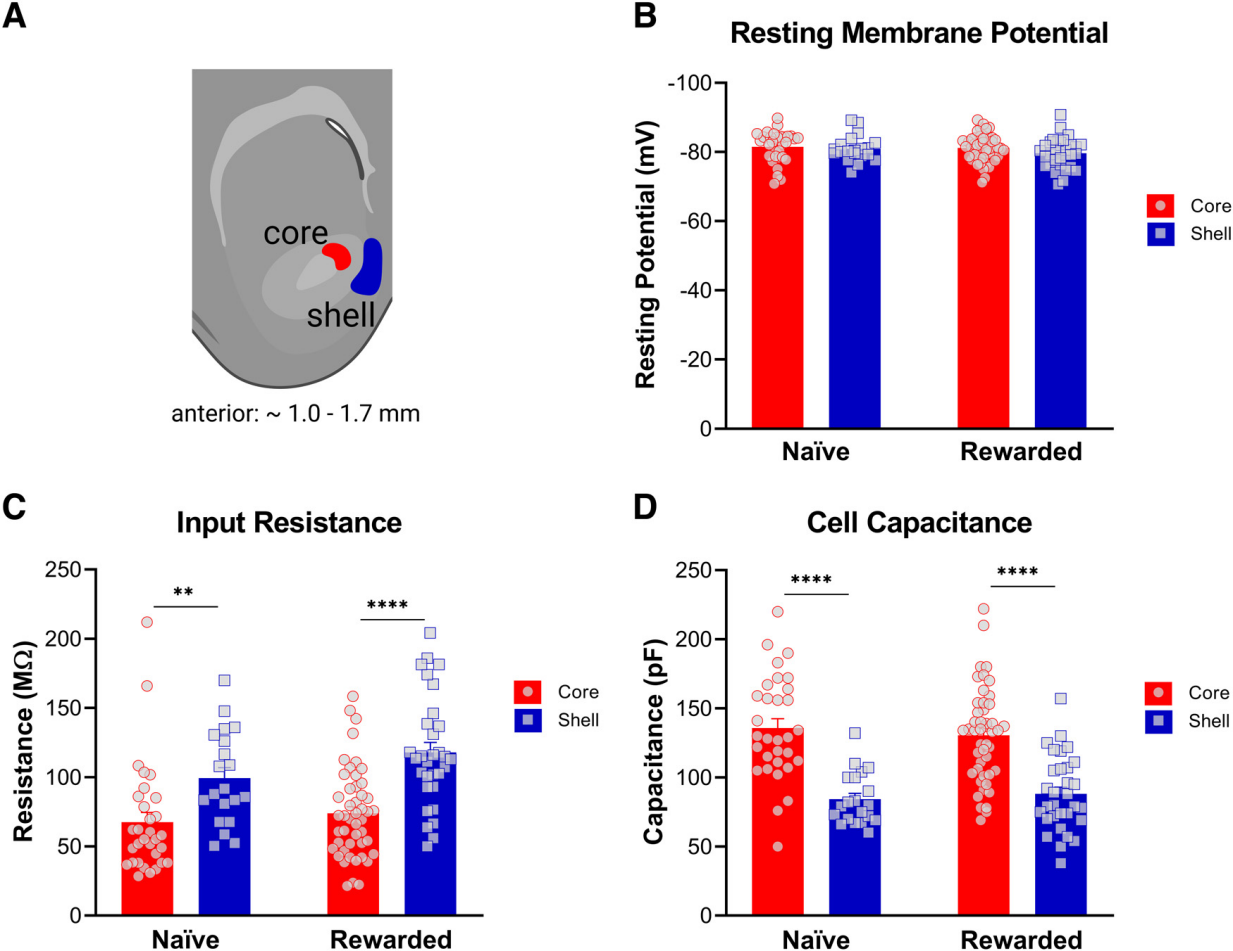
Nucleus accumbens (NAc) medium spiny neurons (MSNs) exhibit distinct passive membrane properties in the core versus shell subregions of both naive and rewarded rats. ***A***, Representative diagram of coronal brain section containing the NAc core (red) and medial shell (blue) subregions (created with BioRender). Whole-cell patch-clamp recordings from medium spiny neurons were obtained from the highlighted areas. ***B***, Resting potential. No significant differences were found in resting potential between NAc core and shell (two-way ANOVA: no main effect of subregion, *p* > 0.05). ***C***, Input resistance. In both naive and rewarded groups, input resistance was significantly greater in NAc shell MSNs compared with core MSNs (two-way ANOVA: main effect of subregion, *p* < 0.001). ***D***, Cell capacitance was significantly lower in NAc shell MSNs compared with core MSNs of both naive and rewarded rats (two-way ANOVA: main effect of subregion, *p* < 0.0001). Significance for Sidak’s *post hoc* test is shown as **p < 0.01, ****p < 0.0001. Each data point represents a single cell. Data are presented as mean ± SEM. Please refer to Table 2 for complete statistical report.

To characterize the intrinsic excitability properties of MSNs in the NAc core and shell, whole-cell electrophysiological recordings were performed in rat brain slices (see Table 1 for summary of data). To confirm the reliability of our findings across subregions, we recorded not only from naive rats, but also from rats that underwent six sessions of unpaired cue/reward exposures (Fig. 1). Electrophysiological analysis revealed differences in passive membrane properties between the two subregions in both naive and rewarded rats (Fig. 2). Input resistance was significantly greater in NAc shell MSNs compared with core MSNs (two-way ANOVA: main effect of subregion, *p* < 0.001; see Table 2 for full statistical report). Consistent with this, cell capacitance was significantly lower in NAc shell MSNs compared with core MSNs of both naive and rewarded rats (two-way ANOVA: main effect of subregion, *p* < 0.0001; see Table 2 for full statistical report). No significant differences were found in resting potential between NAc core and shell MSNs (two-way ANOVA: no main effect of subregion, *p* > 0.05; see Table 2 for full statistical report).

**Table 1:** Electrophysiological passive and active properties of medium spiny neurons in the core and shell of nucleus accumbens of naive and rewarded animals

	Naive		Statistics	Rewarded		Statistics
	Core	Shell	*t*/*F*, *p*	Core	Shell	*t*/*F*, *p*
Passive membrane properties						
Resting membrane potential, mV	−81.5 ± 0.8 (31)	−80.0 ± 0.8 (20)	0.48, 0.86	−81.2 ± 0.6 (47)	−79.7 ± 0.8 (31)	1.60, 0.21
Cell capacitance, pF	136 ± 7 (31)	84 ± 4 (20)	5.66, <0.0001	130 ± 5 (47)	88 ± 5 (31)	5.79, <0.0001
Input resistance, MΩ	67 ± 7 (31)	99 ± 8 (20)	3.07, 0.0053	74 ± 5 (47)	118 ± 7 (31)	5.23, <0.0001
Active membrane properties						
V/I curve (−500 to 0 pA)	−12.0 ± 0.3 (15)	−17.2 ± 0.4 (11)	55.8, 3.61^−13^	−12.7 ± 0.2 (33)	−17.7 ± 0.3 (21)	227, 8.66–47
V/I curve (0 to +100 pA)	5.4 ± 0.5 (15)	10.8 ± 0.6 (11)	40.4, 4.11^−9^	6.1 ± 0.3 (33)	9.8 ± 0.4 (21)	47.3, 4.65^−11^
Sag ratio at −500 pA, mV	1.028 ± 0.003 (15)	1.053 ± 0.005 (11)	4.32, <0.0001	1.029 ± 0.002 (33)	1.053 ± 0.004 (21)	5.85, <0.0001
Number of spikes, AP#	3.2 ± 0.2 (31)	6.7 ± 0.3 (20)	72.8, 8.57^−17^	4.5 ± 0.2 (47)	6.8 ± 0.2 (31)	66.6, 8.93^−16^
Firing frequency, Hz	8.1 ± 0.4 (31)	14.5 ± 0.6 (20)	81.7, 1.48^−18^	9.6 ± 0.4 (47)	14.9 ± 0.4 (31)	84.1, 2.25^−19^
Current to threshold, pA	966 ± 58 (29)	737 ± 45 (20)	2.73, 0.014	943 ± 52 (43)	643 ± 36 (29)	4.32, <0.0001
AP threshold, mV (*)	−44 ± 1.5 (29)	−39.5 ± 1 (20)	2.37, 0.038	−44 ± 1 (43)	−39 ± 1 (29)	2.85, 0.010
Δ RMP/AP threshold, mV (Δb)	36 ± 1.6 (29)	41 ± 1.6 (20)	2.21, 0.056	37 ± 1 (43)	40 ± 1.6 (29)	1.64, 0.20
AP amplitude, mV (▾)	53 ± 1 (29)	50 ± 1 (20)	1.23, 0.39	53 ± 1 (43)	50 ± 1 (29)	2.01, 0.090
Δ RMP/AP amplitude, mV (Δa)	134 ± 1.6 (29)	131 ± 2 (20)	1.01, 0.53	134 ± 1 (43)	128 ± 1.5 (29)	2.64, 0.018
Δ AP threshold/AP amplitude, mV (Δc)	97 ± 2 (29)	90 ± 2 (20)	2.20, 0.059	97 ± 2 (43)	88 ± 2 (29)	2.94, 0.0078
AP halfwidth, ms (↔)	0.66 ± 0.01 (29)	0.68 ± 0.02 (20)	0.76, 0.70	0.68 ± 0.02 (43)	0.70 ± 0.02 (29)	0.62, 0.79

Table lists mean ± SEM (sample size) for passive and active properties of core and shell MSNs for both naive and rewarded rats. Statistics for core versus shell comparisons were obtained from Sidak’s *post hoc* test (*t* value, *p* value) and mixed-effects model planned comparisons (*F* value, *p* value). Main effects and interactions are detailed in Table 2.

**Table 2:** Full statistical report for electrophysiological passive and active properties of medium spiny neurons in the core and shell of nucleus accumbens of naive and rewarded animals

	Effects	*F*, df, *p*	*Post hoc* Comparison	*t*/*F*, df, *p*
Figure 2				
2*B*, resting membrane potential	Subregion (no main effect)^*a*^	*F*_(1,125)_ = 1.90, *p* = 0.17	-	-
2*C*, input resistance	Subregion (main effect)^*a*^	*F*_(1,125)_ = 32.2, *p* < 0.0001	Naive: core vs shell^*c*^ Rewarded: core vs shell^*c*^	*t*_(125)_ = 3.067, *p* = 0.0053 *t*_(125)_ = 5.232, *p* < 0.0001
2*D*, cell capacitance	Subregion (main effect)^*a*^	*F*_(1,125)_ = 64.6, *p* < 0.0001	Naive: core vs shell^*c*^ Rewarded: core vs shell^*c*^	*t*_(125)_ = 5.661, *p* < 0.0001 *t*_(125)_ = 5.789, *p* < 0.0001
Figure 3				
3*C*, voltage/current curve Hyperpolarizing: −500–0 pA Depolarizing: 0–100 pA	Subregion (main effect)^*b*^ Current injection (main effect)^*b*^ Subregion × current injection (interaction)^*b*^ Subregion (main effect)^*b*^ Current injection (main effect)^*b*^ Subregion × current injection (interaction)^*b*^	*F*_(1,1596)_ = 224, *p* = 1.48 × 10^−47^ *F*_(20,1596)_ = 109, *p* = 1.02 × 10^−281^ *F*_(20,1793)_ = 1.68, *p* = 0.03 *F*_(1,375)_ = 86.8, *p* = 1.06 × 10^−18^ *F*_(4,375)_ = 142, *p* = 9.26 × 10^−74^ *F*_(4,375)_ = 11.7, *p* = 6.17 × 10^−9^	Naive: core vs shell^*b*^ Rewarded: core vs shell^*d*^ Naive: core vs shell^*b*^ Rewarded: core vs shell^*d*^	*F*_(1,504)_ = 55.8, *p* = 3.61 × 10^−13^ *F*_(1,1092)_ = 227, *p* = 8.66 × 10^−47^ *F*_(1,117)_ = 40.4, *p* = 4.11 × 10^−9^ *F*_(1,258)_ = 47.3, *p* = 4.65 × 10^−11^
3*E*, sag ratio	Subregion (main effect)^*a*^	*F*_(1,76)_ = 47.6, *p* < 0.0001	Naive: core vs shell^*c*^ Rewarded: core vs shell^*c*^	*t*_(76)_ = 4.32, *p* < 0.0001 *t*_(76)_ = 5.86, *p* < 0.0001
Figure 4				
4*C*, number of spikes	Subregion (main effect)^*b*^ Current injection (main effect)^*b*^ Subregion × current injection (interaction)^*b*^	*F*_(1,1793)_ = 137, *p* = 1.34 × 10^−30^ *F*_(14,1793)_ = 89.4, *p* = 2.17 × 10^−194^ *F*_(14,1793)_ = 2.55, *p* = 0.001	Naive: core vs shell^*b*^ Rewarded: core vs shell^*d*^	*F*_(1,706)_ = 72.8, *p* = 8.57 × 10^−17^ *F*_(1,1087)_ = 66.6, *p* = 8.93 × 10^−16^
4*D*, firing frequency	Subregion (main effect)^*b*^ Current injection (main effect)^*b*^ Group (main effect)^*b*^ Subregion × current injection (interaction)^*b*^	*F*_(1,1793)_ = 162, *p* = 9.17 × 10^−36^ *F*_(14,1793)_ = 97.7, *p* = 8.23 × 10^−209^ *F*_(1,1793)_ = 4.09, *p* = 0.043 *F*_(14,1793)_ = 2.94, *p* = 0.0002	Naive: core vs shell^*b*^ Rewarded: core vs shell^*d*^ Core: Naive vs rewarded^d^	*F*_(1,706)_ = 81.7, *p* = 1.48 × 10^−18^ *F*_(1,1087)_ = 84.1, *p* = 2.25 × 10^−19^ *F*_(1,1097)_ = 7.38, *p* = 0.007
Figure 5				
5*C*, current to threshold	Subregion (main effect)^*a*^	*F*_(1,117)_ = 23.6, *p* < 0.0001	Naive: core vs shell^*c*^ Rewarded: core vs shell^*c*^	*t*_(117)_ = 2.73, *p* = 0.014 *t*_(117)_ = 4.32, *p* < 0.0001
5*D*, threshold potential	Subregion (main effect)^*a*^	*F*_(1,117)_ = 13.3, *p* = 0.0004	Naive: core vs shell^*c*^ Rewarded: core vs shell^*c*^	*t*_(117)_ = 2.37, *p* = 0.038 *t*_(117)_ = 2.86, *p* = 0.01
Table 1 (not in figure)				
Δ RMP/AP threshold	Subregion (main effect)^*a*^	*F*_(1,117)_ = 7.59, *p* = 0.0068	Naive: core vs shell^*c*^ Rewarded: core vs shell^*c*^	*t*_(117)_ = 2.22, *p* = 0.056 *t*_(117)_ = 1.64, *p* = 0.20
AP amplitude	Subregion (main effect)^*a*^	*F*_(1,117)_ = 5.0, *p* = 0.028	Naive: core vs shell^*c*^ Rewarded: core vs shell^*c*^	*t*_(117)_ = 1.23, *p* = 0.39 *t*_(117)_ = 2.01, *p* = 0.090
Δ RMP/AP amplitude	Subregion (main effect)^*a*^	*F*_(1,117)_ = 6.07, *p* = 0.015	Naive: core vs shell^*c*^ Rewarded: core vs shell^*c*^	*t*_(117)_ = 1.00, *p* = 0.53 *t*_(117)_ = 2.64, *p* = 0.018
Δ AP threshold/AP amplitude	Subregion (main effect)^*a*^	*F*_(1,117)_ = 12.7, *p* = 0.0005	Naive: core vs shell^*c*^ Rewarded: core vs shell^*c*^	*t*_(117)_ = 2.20, *p* = 0.059 *t*_(117)_ = 2.94, *p* = 0.0078
AP halfwidth	Subregion (no main effect)^*a*^	*F*_(1,117)_ = 0.96, *p* = 0.33	-	-

Table is organized by figures and lists analyses performed, main effects and interactions, as well as *post hoc* and planned comparisons for group and subregion effects.

a Two-way ANOVA

b Mixed-effects model

c Sidak’s

d Planned comparison

10.1523/ENEURO.0432-22.2023.f1-1 Extended Data Figure 1-1 Individual behavioral responses of Rewarded rats during random lever presentations. Number (***A***) and latency (***B***) of lever presses (black) and magazine entries (gray) during the 10-s lever presentation for each rat in the Rewarded group across all six training sessions. Rats exhibited significantly greater number of magazine entries than lever presses (mixed-effects model: behavioral response × session interaction, *p* < 0.0001) as well as lower latency (mixed-effects model: behavioral response × session interaction, *p* < 0.0001) for magazine entries than lever presses across all six sessions. Behavioral responses are consistent with absence of predictive learning about the lever cue and an increase in magazine entries due to unexpected reward deliveries. Significance for mixed-effect model interaction is shown as ****p < 0.0001. Download Figure 1-1, TIF file.

### MSNs in the NAc shell exhibit greater sag ratios and larger changes in membrane potential in response to current injections than core MSNs of both naive and rewarded rats

**Figure 3.**
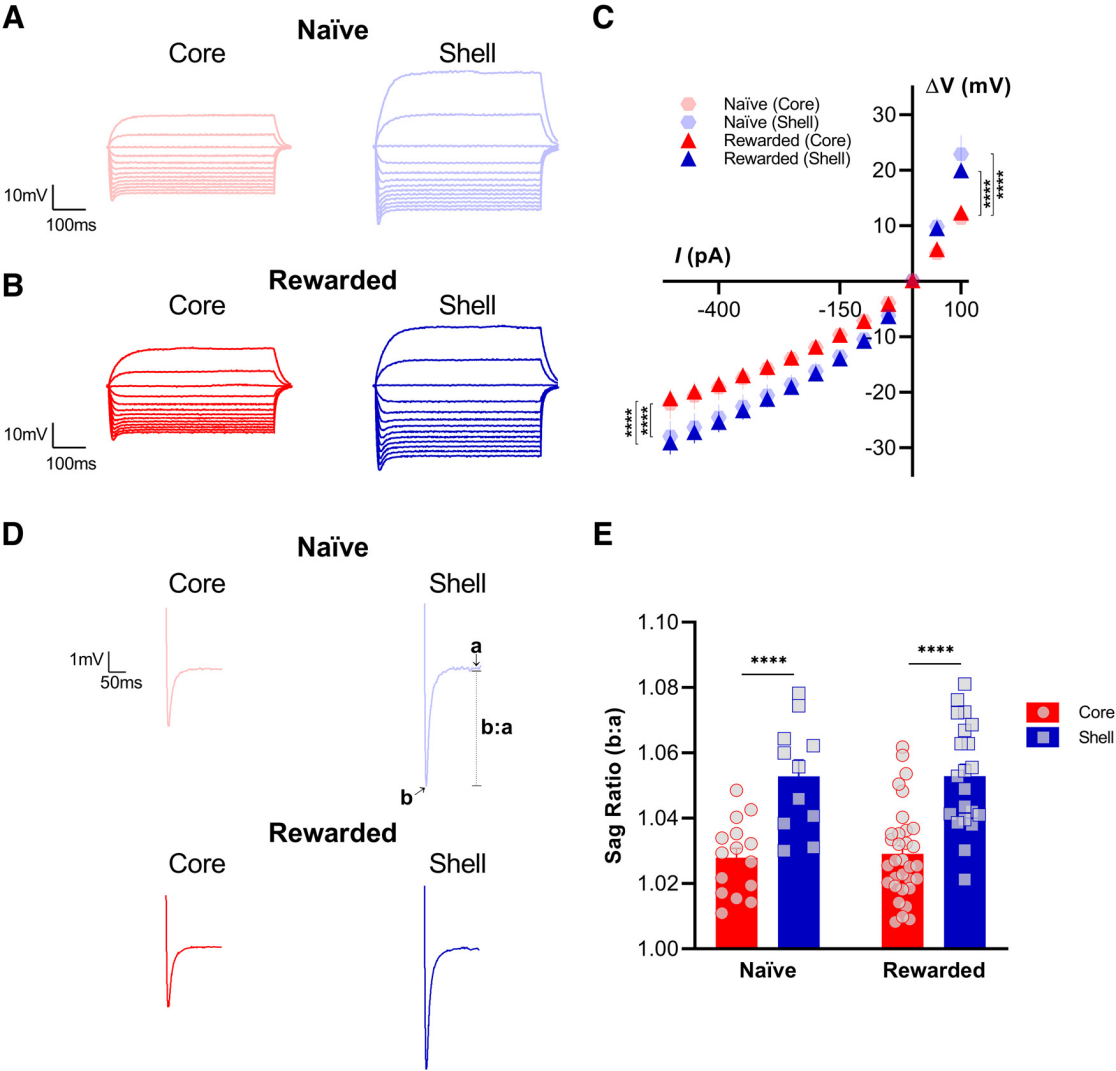
Nucleus accumbens (NAc) medium spiny neurons (MSNs) in the shell exhibit greater sag ratios and larger changes in membrane potential in response to current injections than core MSNs of both naive and rewarded rats. Representative voltage response traces from current-clamp recordings of MSNs in NAc core (left) and shell (right) slices from (***A***) naive and (***B***) rewarded rats. Current injection step protocol ranged from −500 to +100 pA and is shown here in 50-pA increments. ***C***, Voltage/current (V/I) relationship curve is significantly different between NAc shell and core MSNs of both naive and rewarded rats. The same hyperpolarizing (−500–0 pA) and depolarizing (0–100 pA) current injection steps consistently elicited a greater change in membrane potential in the shell versus in the core, causing a significant shift of the V/I curve (hyperpolarizing: mixed-effects model: main effect of subregion, *p* < 0.0001; depolarizing: mixed-effects model: main effect of subregion, *p* < 0.0001). ***D***, Representative current-clamp recordings of voltage sag response to a −500-pA current injection from MSNs of NAc core (left) and shell (right) slices from naive (top) and rewarded (left) rats. Traces are shown as the average from all cells for each group and subregion. ***E***, Sag ratio (b:a) was obtained by dividing the peak voltage response to a −500-pA current injection (b) over the steady-state response 200 ms from the onset of stimulation (a). In both naive and rewarded rats, MSNs in the NAc shell showed a greater sag ratio, representative of a larger sag, compared with MSNs in the NAc core (two-way ANOVA: main effect of subregion, *p* < 0.0001). Significance for mixed-effect model planned comparison (***C***) and Sidak’s *post hoc* test (***E***) is shown as ****p < 0.0001. Each data point represents a single cell. Data are presented as mean ± SEM. Please refer to Table 2 for complete statistical report.

To investigate potential subregional differences in active intrinsic excitability properties between the NAc core and shell, we analyzed membrane potential responses of MSNs to current injections from −500 to +100 pA in 25-pA increments, generating a voltage/current relationship curve. Consistent with our findings for passive membrane properties, NAc MSNs in the shell exhibited larger responses to current injections compared with responses recorded in core MSNs of both naive and rewarded rats (Fig. 3). The same hyperpolarizing (−500–0 pA) and depolarizing (0–100 pA) current injection steps consistently elicited a greater change in membrane potential in the shell than in the core, causing a significant shift of the V/I curve for both the naive and rewarded groups curve (hyperpolarizing: mixed-effects model: main effect of subregion, *p* < 0.0001; depolarizing: mixed-effects model: main effect of subregion, *p* < 0.0001; see Table 2 for full statistical report). In addition, we tested whether MSNs in the NAc core versus shell showed a significant difference in their voltage sag response to a −500-pA current injection (Fig. 3). To examine this further, a sag ratio was calculated by dividing the peak voltage response to −500 pA over the steady-state response 200 ms from the onset of stimulation. In both naive and rewarded rats, MSNs in the NAc shell showed a greater sag ratio, representative of a larger sag, compared with MSNs in the NAc core (two-way ANOVA: main effect of subregion, *p* < 0.0001; see Table 2 for full statistical report).

### NAc MSNs in the shell exhibit higher firing frequencies than core MSNs of both naive and rewarded rats as well as differences in action potential properties

**Figure 4.**
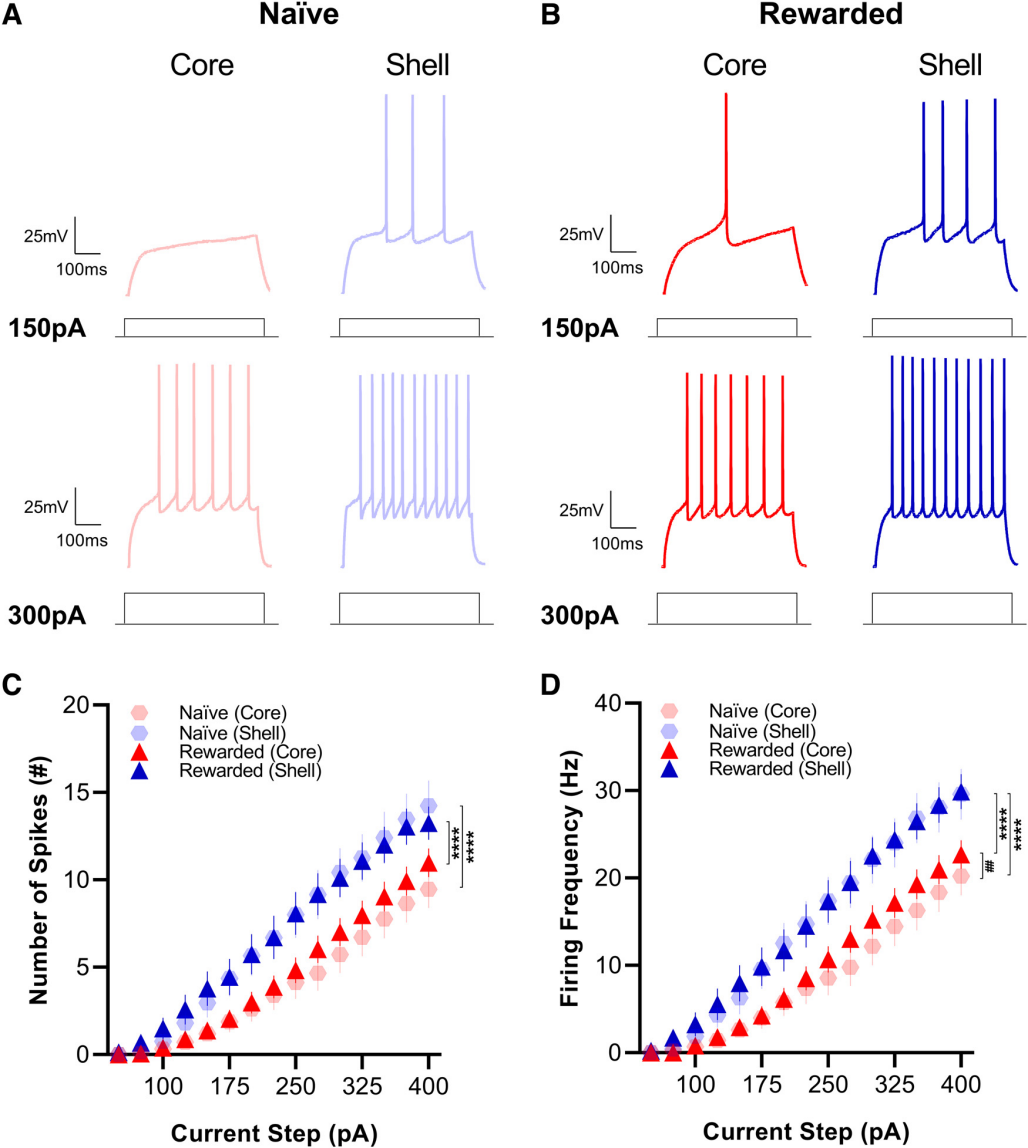
Medium spiny neurons (MSNs) located in the shell exhibit greater excitability than core MSNs of both naive and rewarded rats. Representative traces of current-clamp recordings from MSNs in NAc core (left) and shell (right) slices of (***A***) naive and (***B***) rewarded rats in response to 150-pA (top) and 300-pA (bottom) current injections. ***C***, Number of spikes. MSNs in the NAc shell showed a higher number of spikes compared with MSNs in the NAc core in response to current injection steps of the same magnitude (mixed-effects model: main effect of subregion, *p* < 0.0001). ***D***, Firing frequency. Similarly, MSNs in the NAc shell showed greater firing frequency compared with MSNs in the NAc core in response to current injection steps of the same magnitude (mixed-effects model: main effect of subregion, *p* < 0.0001). MSNs in the core of naive rats also had lower firing frequency compared with those in the rewarded group (mixed-effects model: main effect of group; *p* < 0.05). Significance for mixed-effect model planned comparison is shown as ****p < 0.0001, ##*p* < 0.01. Data are presented as mean ± SEM. Please refer to Table 2 for complete statistical report.

To further examine the intrinsic excitability of core and shell MSNs of naive and rewarded rats, we measured firing rates in response to current injections from +50 to +400 pA in 25-pA increments. We found that in response to current injection steps of the same magnitude, MSNs in the shell had a significantly higher number of spikes (mixed-effects model: main effect of subregion, *p* < 0.0001; see Table 2 for full statistical report) and greater firing frequency (mixed-effects model: main effect of subregion, *p* < 0.0001) compared with MSNs in the NAc core in both the naive and rewarded group (Fig. 4). In addition, MSNs in the core of naive rats showed a lower firing frequency compared with those in the rewarded group (mixed-effects model: main effect of group; *p* < 0.05; see Table 2 for full statistical report) suggesting that the reward exposure experience may induce a slight increase in the excitability of MSNs in the NAc core.

**Figure 5.**
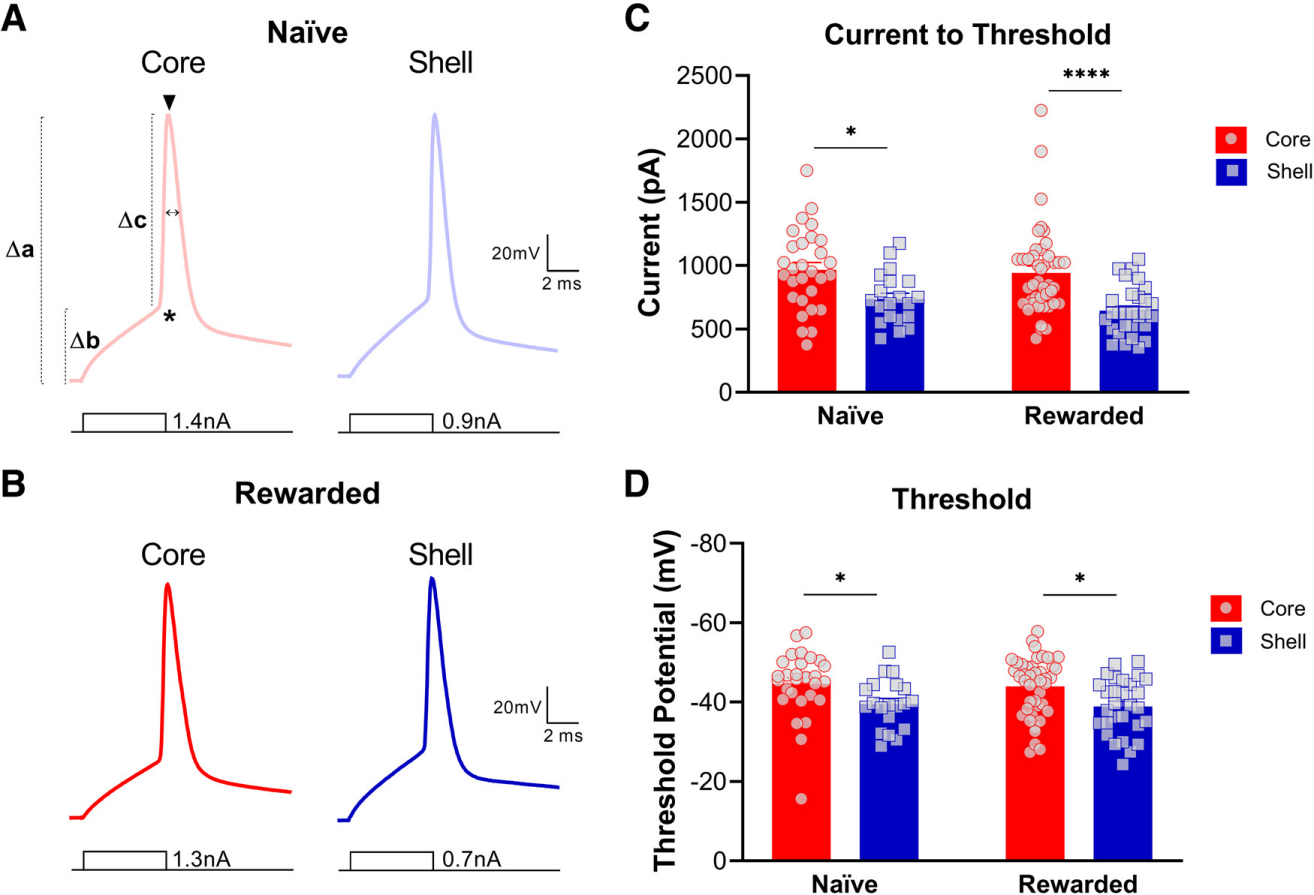
Nucleus accumbens (NAc) medium spiny neurons (MSNs) exhibit distinct action potential properties in the core versus shell subregions of both naive and rewarded rats. Representative single action potential traces of current-clamp recordings from MSNs in NAc core (left) and shell (right) slices of (***A***) naive and (***B***) rewarded rats. Symbols illustrated on A (left) represent the following: * = AP threshold (mV), Δb = Δ RMP/AP threshold (mV), ▾ = AP amplitude (mV), Δa = Δ RMP/AP amplitude (mV), Δc = Δ AP threshold/AP amplitude (mV), ↔ = AP halfwidth (ms). ***C***, Current to threshold: In both naive and rewarded groups, the current necessary to elicit a single action potential was significantly lower in NAc shell MSNs compared with core MSNs (two-way ANOVA: main effect of subregion, *p* < 0.0001). ***D***, Action potential threshold. AP threshold was significantly more depolarized in NAc shell MSNs compared with core MSNs of both naive and rewarded rats (two-way ANOVA: main effect of subregion, *p* < 0.001). Significance for Sidak’s *post hoc* test is shown as *p < 0.05, ****p < 0.0001. Each data point represented a single cell. Data are presented as mean ± SEM. Please refer to Table 2 for complete statistical report.

To study single spike properties in MSNs in the NAc core and shell, neurons received 5-ms current injections in 25-pA increments until a single AP was elicited. Upon analysis, we found significant subregional differences in both naive and rewarded groups for most action potential properties (Fig. 5; Tables 1, 2). Consistent with the results above, the current necessary to elicit a single action potential was significantly lower in NAc shell MSNs compared with core MSNs (two-way ANOVA: main effect of subregion, *p* < 0.0001; see Table 2 for full statistical report). Additionally, the AP threshold was significantly more depolarized in NAc shell MSNs compared with core MSNs of both naive and rewarded rats (two-way ANOVA: main effect of subregion, *p* < 0.001; see Table 2 for full statistical report). Main effects of subregion were obtained for Δ RMP/AP threshold, AP amplitude, Δ RMP/AP amplitude, and Δ AP threshold/AP amplitude, which all indicate a generally larger action potential threshold and smaller AP amplitude for shell MSNs compared with core MSNs. No significant differences were found for AP halfwidth (see Tables 1, 2 for statistical report).

## Discussion

Our data show that MSNs in the core and shell differ in their passive and active membrane properties. Overall, MSNs within the medial NAc shell are significantly more excitable than MSNs in the NAc core. In particular, we found that shell MSNs had greater input resistance and lower cell capacitance compared with core MSNs. We also found a significant difference in the voltage/current relationship, with shell MSNs consistently showing a greater deflection in membrane potential in response to hyperpolarizing and depolarizing current injections. This was accompanied by a greater sag ratio for shell MSNs, which is a measure of the hyperpolarization-activated cation current, or Ih (Pape, 1996; Robinson and Siegelbaum, 2003). As expected, we found that shell MSNs exhibited a greater number of action potentials in response to current injection steps as well as greater firing frequency compared with core MSNs. The current necessary to induce a single action potential was also lower for shell MSNs. Interestingly, we found subregional differences between the action potential properties. Core MSNs had a significantly more hyperpolarized action potential threshold, and overall, a larger action potential amplitude as measured from zero, resting, and the threshold potential.

Since behavioral and environmental enrichment have been previously reported to induce changes and adaptations in neuronal excitability of pyramidal neurons in the hippocampus (Malik and Chattarji, 2012; Valero-Aracama et al., 2015) as well as in MSNs in the NAc (Scala et al., 2018), we wanted to determine how stable subregional differences between core and shell would be regardless of the behavioral experience of the rats. We therefore recorded from core and shell MSNs of brain slices from both “naive” rats and “rewarded” rats that underwent six sessions of unpaired cue/reward exposures. Subregional differences in intrinsic excitability were highly consistent between the naive and rewarded groups. There were no significant differences in the number of action potentials, voltage/current relationship, or input resistance. However, naive animals had a lower firing frequency of core MSNs compared with rewarded animals. This suggests that reward exposures may have caused a slight increase in the excitability of core MSNs, consistent with previously reported *in vivo* changes in response to food rewards during learning tasks (Carelli, 2002; Day et al., 2006). It is worth noting that a resting period of one to three weeks following the unpaired CS-US task was imposed for the rewarded group rats. The purpose was to allow enough time to focus on intrinsic and not altered excitability differences between the rewarded and naive groups. We acknowledge that the impact on excitability of the reward exposures could have lingered regardless of the resting period, but the subtle difference reported between the two groups makes such lingering effects seem unlikely. Therefore, we focus on highlighting the finding that subregional excitability differences of core and shell MSNs are stable and not very sensitive to simple behavioral experiences. Another important note is that though these subregional differences were very pronounced in both groups, it remains unknown whether they would be present in female rats, as we only studied male rats. Previous studies have reported excitability differences in the NAc of male and female rats that are particularly vulnerable to sex hormones (Cao et al., 2018; Proaño et al., 2018). Therefore, it is possible that this could translate to differential core versus shell subregional excitability in female rats.

The reported findings are consistent with known anatomic and morphologic differences between core and shell MSNs. Some studies have shown that neurons in the shell have significantly fewer dendritic arbors, branch segments, terminal segments, and lower spine densities than those in the core (Meredith et al., 1992; O’Donnell and Grace, 1993; Forlano and Woolley, 2010; Wissman et al., 2011). These morphologic differences result in some shell MSNs having up to ∼50% less surface area than core MSNs (Meredith et al., 1992). A lower surface area can result in significantly lower cell capacitance and consequently greater input resistance, providing a direct link between morphologic and physiological properties. These marked differences in input resistance may be the primary cause of the greater excitability of shell MSNs compared with core MSNs. Although many electrophysiological studies in MSNs have measured the sag index (Belleau and Warren, 2000; Dehorter et al., 2009; Proaño and Meitzen, 2020), this is the first study reporting a subregional difference in sag between core and shell MSNs. Greater hyperpolarized responses to negative current injections could be activating more hyperpolarization-activated cation channels (HCN), which are known to be expressed in the NAc (Uchimura et al., 1990; Monteggia et al., 2000; Notomi and Shigemoto, 2004; Santos-Vera et al., 2013), thus resulting in a greater sag response in shell MSNs (Robinson and Siegelbaum, 2003). Studies have identified striatal cholinergic neurons whose spontaneous tonic firing is regulated by Ih and is sensitive to dopaminergic modulation (Bennett et al., 2000; Deng et al., 2007). Interestingly, the mRNA and protein expression of HCN subunits in the NAc is thought to be very low (Monteggia et al., 2000; Santos-Vera et al., 2013), and the role of the Ih current in the MSN neuronal population remains largely unknown (Uchimura et al., 1990; Inoue et al., 2012). Nonetheless, cocaine sensitization increases the expression of HCN_2_ in the NAc without affecting the surface/intracellular ratio (Santos-Vera et al., 2013, 2019), and inhibition of HCN in the NAc significantly reduces methamphetamine self-administration (Cao et al., 2016). Therefore, Ih current in the NAc may modulate neuronal excitability and network dynamics, thereby modulating the reinforcing effect of drugs.

Our data are consistent with previous electrophysiological studies in mice, which also reported shell MSNs to exhibit greater input resistance and overall greater number of action potentials compared with core MSNs (Kourrich and Thomas, 2009). In rats, direct core versus shell intrinsic excitability comparisons have been somewhat contradictory. Consistent with our findings and those found in mice, one study reported that core MSNs had lower input resistance as well as a more negative resting membrane potential compared with shell MSNs (Pennartz et al., 1992). Conversely, another study found that overall core and shell MSNs had very similar passive membrane properties, and that shell MSNs were less excitable than core MSNs (O’Donnell and Grace, 1993). These apparent inconsistencies could be because of methodological differences in slice electrophysiology. Nonetheless, some anatomic studies have also reported contrasting findings in the differences between core and shell MSN morphology (Záborszky et al., 1985). Interestingly, it seems that a medial to lateral gradient in spine density and branching exists within the shell. Neurons in the lateral portion of the shell more closely resemble the morphology of neurons in the core (Meredith et al., 1992), meaning that if these physiological differences are linked to morphologic differences, the location within each subregion is crucial for detecting specific differences in core versus shell intrinsic excitability properties. For example, this could suggest that the intrinsic excitability properties of MSNs within the lateral portion of the NAc shell could more closely resemble those from core MSNs, which could be further explored in subsequent studies. This could at least partially explain incongruent findings regarding core versus shell neuronal morphology and physiology.

In the core and shell of the NAc, MSNs differ not only in their morphology, but also in their distinctive patterns of connectivity with mesencephalic regions (Groenewegen and Russchen, 1984; Deutch et al., 1988; Heimer et al., 1991; Berendse et al., 1992; Meredith et al., 1995; Zahm, 1999; Breton et al., 2019). These anatomic differences are accompanied by heterogeneity in dopamine D_1_ and D_2_ receptor expression as well as dopamine levels and utilization in the core and shell subregions. Overall, several studies have found that D_1_ receptors outnumber D_2_ receptors in the shell, while in the core D_2_ receptors are more abundant (Bardo and Hammer, 1991; Lu et al., 1998; Hasbi et al., 2020). In addition, tyrosine hydroxylase immunoreactivity indicates that the shell is more densely innervated by dopaminergic terminals (Zahm, 1992), and dopamine levels are greater in the shell compared with the core (Deutch and Cameron, 1992). These differences in D_1_ and D_2_ receptor expression can functionally impact neuronal excitability (Deroche et al., 2020). For example, activation of D_1_-like receptors of MSNs can increase MSN depolarization by inhibiting Kir-channel K^+^ currents (Podda et al., 2010) and by enhancing L-type Ca^2+^ currents (Hernández-López et al., 1997). Although activation of D_2_ in MSNs of the NAc can also increase depolarization of the resting membrane potential by decreasing K^+^ leak currents, it has been shown to significantly decrease action potential firing via A-type K^+^ currents (Perez et al., 2006) and L-type Ca^2+^ currents (Hernandez-Lopez et al., 2000). Therefore, morphologic differences between core and shell MSNs might not be the only cause for the subregional differences in membrane properties, but distinctive modulation from the dopaminergic system may also impact neuronal excitability. Substances like cocaine (Kourrich and Thomas, 2009), morphine (Madayag et al., 2019), and nicotine (Nisell et al., 1997) have also been found to have distinctive impacts on core and shell MSN intrinsic excitability and synaptic activity, providing further evidence of physiological and functional differences in neuronal properties.

The NAc is a critical structure of the motive circuit as it converges both cortical and subcortical information during associative learning to ultimately process and regulate motivated behaviors (Day and Carelli, 2007; Floresco, 2015; Salgado and Kaplitt, 2015). The intrinsic excitability state of MSNs – how sensitive neurons are to changes in potential in response to input stimuli, can heavily influence how the NAc encodes and relays reward information (O’Donnell and Grace, 1996; Nicola et al., 2000). The subregional differences in intrinsic excitability reported here provide a potential physiological link to the different morphologic and anatomic characteristics of core and shell MSNs. This can be used to further inform investigations of their distinct roles in reward learning (Zahm, 1999; Ito and Hayen, 2011; Saddoris et al., 2015; West and Carelli, 2016) and in generating problematic behavioral responses linked to disorders like addiction (Di Chiara, 2002; Ito et al., 2004), anxiety (Dutta et al., 2021), and impulsivity (Pattij et al., 2007; Feja et al., 2014).

## Synthesis

Reviewing Editor: Matthew Grubb, King’s College London Decisions are customarily a result of the Reviewing Editor and the peer reviewers coming together and discussing their recommendations until a consensus is reached. When revisions are invited, a fact-based synthesis statement explaining their decision and outlining what is needed to prepare a revision will be listed below. The following reviewer(s) agreed to reveal their identity: Jessica Loweth. Note: If this manuscript was transferred from JNeurosci and a decision was made to accept the manuscript without peer review, a brief statement to this effect will instead be what is listed below.

Reviewer 1:

In this study the authors compared the passive and active intrinsic properties between the NAc medial shell and core MSNs in adult male rats using whole-cell patch clamp recordings. Using naïve rats and rewarded rats (which receive non-contingent cue/reward exposures), they showed that NAc shell MSNs are more excitable than core MSNs in both the naïve and rewarded rats. Moreover, the authors found that core MSNs showed a higher excitability in the rewarded group compared to those in naïve rats. Although sex or cell-type specificity in intrinsic properties of MSNs in NAc is not addressed, this manuscript provides a good addition to the current knowledge on electrophysiological properties of NAc shell and core MSN. In general, the electrophysiology experiments are well conducted and analyzed. The Discussion is detailed and well written.

Below are my questions/comments for improving the manuscript:

1. In the second paragraph of the Introduction, the authors cite Planert et al 2013 for differences between D1 and D2 MSN of the NAc. This citation is not appropriate as experiments were done in dorsal striatum. I suggest citing M.A. Deroche et al J Neurosci 2020 instead. And, if there are any other citations here that refer to dorsal striatal MSN rather than NAc, please replace with NAc citations.

2. The authors should show behavioral data for “rewarded” rats in Figure 1 to clarify how much individual variability exists for this group. Also, please explain why electrophysiological recordings were performed 1-3 weeks after the reward period and acknowledge in the text that cellular changes produced by this reward paradigm may not persist after rats spend 1-3 weeks in their home cages.

3. The best control group would be a non-rewarded group that undergoes the experimental procedure but receives cue but no associated food. This limitation should be acknowledged.

4. Among the subregions of NAc shell, the medial shell of the NAc was the focus of this study. Justification for focusing on the medial shell will be helpful. Give the discussion on the relationship between morphology and physiology, do the authors expect the electrophysiological properties of lateral shell MSN to resemble core MSN? Since NAc lateral shell is also implicated in reward and motivated behavior, please mention anything that is known about intrinsic properties of NAc lateral shell MSN.

5. On p. 22 of the pdf the authors state that medial shell MSNs are primarily innervated by mesostriatal dopaminergic projections while those in the core receive mainly nigrostriatal dopaminergic innervation. This statement should be rewritten to be more accurate and specific. While there are differences in connectivity between midbrain subregions and accumbens subregions, the VTA projects to both shell and core (whereas the current statement implies core is mainly innervated by SN). A more recent paper that can be cited and which also contains information about the transmitter of VTA-NAc projections is JC Breton et al J Comp Neurol 2019.

6. Figure 4 C, D: Including both the number of spikes and firing frequency seems redundant as they should be inter-dependent. However, while firing frequency in core MSN shows a significant difference between naïve rats and rewarded rats (reflected in figure legend to Fig 4D and table 2), spike number does not differ between naive and rewarded rats (although there is a trend apparent in Fig 4C of a magnitude similar to that found to be significant for firing frequency in Fig 4D). Since the reward effect on firing frequency looks subtle and this is the only difference after reward, the authors should add additional analysis of data or detailed explanation to make clear the effects of reward on excitability in core MSN. Also, it would help to add a symbol indicating a significant naïve vs rewarded difference for core MSN in Fig. 4C.

Minor

1. Figure 3A-C, 4A-B, 5A-B: Although the RMP is comparable between shell and core MSN, please clarify whether the current clamp recordings were conducted at the equalized membrane potentials by injecting currents. It would be good to indicate the membrane potential for each sweep in the figures.

2. Methods: Please clarify whether liquid junction potential is corrected.

3. Methods: It would be helpful if the authors report what percent of MSN show depolarization block and if there is any difference between shell and core.

4. page 12 line 6, Figure 3 legend: typo paA naïve pA

5. Abstract: A period is before the references in the 4th line and second to last line.

Reviewer 2:

This is a generally well-written manuscript investigating differences in passive membrane properties and intrinsic excitability between neurons in the nucleus accumbens (NAcc) core and shell subregions in both unhandled drug-naïve male rats that remained in their homecages and “rewarded” male rats that underwent unpaired cue/food reward exposures. While many studies have investigated intrinsic excitability of NAcc medium spiny neurons (MSNs) following exposure to drugs of abuse or in models of obesity, stress and depression, less is known about subregional differences in intrinsic membrane excitability in drug-naïve animals. I feel that this is an important gap to address, although the fact that these studies were only conducted in male rats is a shortcoming of the manuscript. I have several comments for the authors to address:

1. I find the last sentence of the Introduction to be confusing, especially following a sentence describing how enrichment can influence intrinsic excitability. I suggest removing/editing this sentence to make it more of a summary sentence about what will be done in the manuscript. Related to this, I feel that, in the framing of the Introduction, the authors could highlight that a strength of the manuscript is that both groups are included in their analyses (drug-naïve controls vs food reward group) rather than making it sound like these are just two different sorts of control groups.

2. In the legend for Figure 1, in the first few sentences - please define each group of rats in parentheses when describing them. Also, please be consistent with the use of homecage naïve or naïve. In Figure 1, the rats are referred to as “naïve” in the arrow but then “homecage naïve” in the timeline and then not specifically called “homecage naïve rats” in the legend.

3. It is not clear to me why the homecage drug-naïve group was not handled for the “entirety of the experiment”. Please clarify why there was not a handled control group (the other group gets handled but also gets food rewards, I am referring to just a handled drug-naïve homecage group) and acknowledge that the lack of handling is distinct from commonly used drug-naïve control groups in the literature. Please also refer to this group as the unhandled homecage drug-naïve group or something similar, as to more accurately describe the group.

4. Please clarify throughout that the reward is a food reward. Reward is such a general term and could be used to describe many different substances.

5. While I understand that including some extra information in the tables is helpful, I feel that some of the information in the tables is redundant with that in the figures and/or not necessary. Much of this can be summarized in the text in a sentence or two. I suggest only putting in the tables information that is directly related to (but not the same as) what is shown in the figures and only very pertinent information. Otherwise, stating no differences were found/data not shown is acceptable for many of these properties.

6. This sentence in the first paragraph of the Discussion is misleading: “Overall, MSNs within the NAc shell, specifically within the medial portion, are significantly more excitable that MSNs in the NAc core.” This makes it sound like analyses were conducted in different areas of the shell. Please rewrite as “MSNs within the medial NAcc shell” and make sure this is consistently done throughout (which it does seem to be in other places).

7. These studies were conducted in male rats, which should be stated more throughout the manuscript and included in the title. In the Introduction, the authors acknowledge that the effects of sex (most of which were conducted in prepubertal females and not adult females, which is an important distinction) and estrous cycle on intrinsic membrane excitability of NAcc MSNs has been previously investigated. However, in the Discussion there is no mention of the fact that females were not included in the current study and no discussion of previous findings conducted in females (either prepubertal or adult females) and how they compare to the current findings. This is a shortcoming of the manuscript in my opinion, as I do not believe that intrinsic membrane properties have been assessed between core and shell in adult female drug-naïve rats (not to mention estrous cycle effects). Please address/acknowledge this.

8. References - Dorris et al 2015a and 2015b are currently the same manuscript. This needs to be fixed.
