# Supplementary figures and images for: Subregional Differences in Medium Spiny Neuron Intrinsic Excitability Properties between Nucleus Accumbens Core and Shell in Male Rats
Source: eNeuro. 2023 May 17;10(5):ENEURO.0432-22.2023. doi: 10.1523/ENEURO.0432-22.2023 (PMC10206613; doi:10.1523/ENEURO.0432-22.2023)

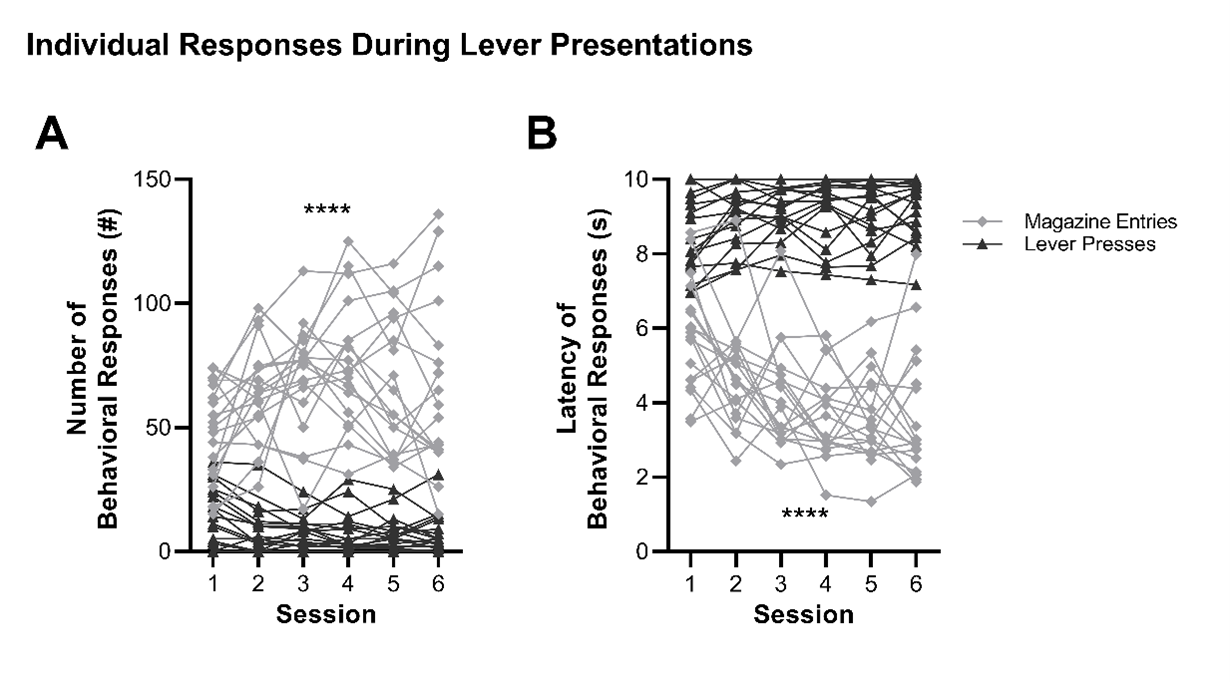

Supplement: Extended Data Figure 1-1 — Individual behavioral responses of Rewarded rats during random lever presentations. Number (A) and latency (B) of lever presses (black) and magazine entries (gray) during the 10-s lever presentation for each rat in the Rewarded group across all six training sessions. Rats exhibited significantly greater number of magazine entries than lever presses (mixed-effects model: behavioral response × session interaction, p < 0.0001) as well as lower latency (mixed-effects model: behavioral response × session interaction, p < 0.0001) for magazine entries than lever presses across all six sessions. Behavioral responses are consistent with absence of predictive learning about the lever cue and an increase in magazine entries due to unexpected reward deliveries. Significance for mixed-effect model interaction is shown as ****p < 0.0001. Download Figure 1-1, TIF file. [file enu-eN-NWR-0432-22-s02.tif]
